# Associations between speech features and phenotypic severity in Treacher Collins syndrome

**DOI:** 10.1186/1471-2350-15-47

**Published:** 2014-04-28

**Authors:** Pamela Åsten, Harriet Akre, Christina Persson

**Affiliations:** 1 TAKO-centre, Lovisenberg Diakonale Hospital, 0440 Oslo, Norway; 2 Institute of Clinical Medicine, University of Oslo, Oslo, Norway; 3 Department of Otorhinolaryngology, Head and Neck Surgery, Oslo University Hospital, Oslo, Norway; 4 Institute of Neuroscience and Physiology, Division of Speech and Language Pathology, The Sahlgrenska Academy, University of Gothenburg, Gothenburg, Sweden

**Keywords:** OMIM 154500, Mandibulofacial dysostosis, Articulation, Nasal resonance, Voice, Intelligibility, Phenotype, Orofacial dysfunction

## Abstract

**Background:**

Treacher Collins syndrome (TCS, OMIM 154500) is a rare congenital disorder of craniofacial development. Characteristic hypoplastic malformations of the ears, zygomatic arch, mandible and pharynx have been described in detail. However, reports on the impact of these malformations on speech are few. Exploring speech features and investigating if speech function is related to phenotypic severity are essential for optimizing follow-up and treatment.

**Methods:**

Articulation, nasal resonance, voice and intelligibility were examined in 19 individuals (5–74 years, median 34 years) divided into three groups comprising children 5–10 years (n = 4), adolescents 11–18 years (n = 4) and adults 29 years and older (n = 11). A speech composite score (0–6) was calculated to reflect the variability of speech deviations. TCS severity scores of phenotypic expression and total scores of Nordic Orofacial Test-Screening (NOT-S) measuring orofacial dysfunction were used in analyses of correlation with speech characteristics (speech composite scores).

**Results:**

Children and adolescents presented with significantly higher speech composite scores (median 4, range 1–6) than adults (median 1, range 0–5). Nearly all children and adolescents (6/8) displayed speech deviations of articulation, nasal resonance and voice, while only three adults were identified with multiple speech aberrations. The variability of speech dysfunction in TCS was exhibited by individual combinations of speech deviations in 13/19 participants. The speech composite scores correlated with TCS severity scores and NOT-S total scores. Speech composite scores higher than 4 were associated with cleft palate. The percent of intelligible words in connected speech was significantly lower in children and adolescents (median 77%, range 31–99) than in adults (98%, range 93–100). Intelligibility of speech among the children was markedly inconsistent and clearly affecting the understandability.

**Conclusions:**

Multiple speech deviations were identified in children, adolescents and a subgroup of adults with TCS. Only children displayed markedly reduced intelligibility. Speech was significantly correlated with phenotypic severity of TCS and orofacial dysfunction. Follow-up and treatment of speech should still be focused on young patients, but some adults with TCS seem to require continuing speech and language pathology services.

## Background

Treacher Collins syndrome (TCS, OMIM 154500) is a rare congenital disorder of craniofacial development affecting 1 in 50 000 live births [1]. The disorder most commonly arises from mutations of the TCOF1 gene encoding for the treacle protein, which is essential for craniofacial development [2]. TCOF1 mutations are inherited in an autosomal dominant manner, but approximately 60% are de novo mutations [1]. Hypoplastic malformations of the ears, zygomatic arch, mandible and pharynx [3-8] due to neuroepithelial cell death [1] have been described in detail. However, reports on the impact of these malformations on speech are few and relate primarily to individuals younger than 20 years of age [9-11]. Extended understanding of speech characteristics in TCS and investigating how these are associated with other features typical of the syndrome is therefore of great interest.

The variability of phenotypic expression of TCS is considerable [12]. The chin is dysplastic and horizontally retracted, resulting in markedly reduced posterior facial height. Pronounced facial convexity is related to mandibular retrognathism causing anterior open bite malocclusion, while lip incompetence is related to retruded lower lip and chin [3,6]. The nasal passages may be obstructed by choanal atresia or stenosis due to maxillary hypoplasia [13]. Pharyngeal hypoplasia at all levels, with the most severe narrowing at the junction of the oro- and hypopharynx near the tongue base, is a primary feature in TCS [8]. Nasal abnormalities and pharyngeal restrictions are assumed to compromise respiration and affect swallowing [3,8]. Compromised respiration at birth has been reported in nearly half of the 47 patients treated by the Australian Craniofacial Unit [10]. Some children may require tracheostomy from early life until the airway restriction can be resolved by mandibular advancement to relieve obstruction on tongue base level [10,14,15].

Isolated cleft palate has been reported in approximately one-third of affected individuals [6,9,11]. Other types of palatal malformations such as submucous clefts, bifid uvula and short palate can occur [9,11]. Complicated airway management may delay palatal repair with an average of one year [10].

Anterior open bite and Class II malocclusion are common findings in children and adolescents with TCS, while Class I and III malocclusion are more rare [10,11]. Orthodontic treatment either alone or supplemented with orthognatic procedures is often necessary to improve occlusion [10].

Variable degrees of auricular deformities and malformations of the middle ear are typical features of TCS [10,12]. Atresia or stenosis of the external auditory canal is common [7,12] and found to be closely related to hypoplastic or absent middle ear ossicles [7]. Conductive hearing loss to a moderate or greater degree has been reported in almost all individuals with TCS, while mixed hearing loss is reported in a small percentage of patients [7,10,11].

A combined retrospective and prospective study of speech comprising 30 children and adolescents affected with TCS has revealed articulation errors in all participants [11]. The articulation errors were assigned to three categories according to the assumed etiology: occlusion, velopharyngeal insufficiency (VPI), or general articulatory/phonologic difficulties. Some of the patients had overlapping errors. Malocclusion was considered the causative factor in a majority of the articulation errors, and was mainly associated with interdentalization of lingual alveolar sibilants. Abnormal nasal resonance, in terms of muffled resonance quality irrespective of palatal anatomy, was observed in 77 percent of participants. This muffled quality was considered to interfere with the perception of hypernasality and voice. Hypernasality was identified in approximately one-third of the patients with cleft palate, while hyponasality occurred in some of the children without clefts. Voice quality was classified as normal in 60 percent of participants.

Another study has reported speech abnormalities in 74 percent of the 46 patients with TCS who were treated at a craniofacial unit from birth to maturity [10]. The abnormalities were associated with size restriction of the nasal passages and oropharynx. Hyponasality was the most common finding, while children with clefts and VPI exhibited hypernasality. Articulation errors were present in approximately.

Previous studies have established that speech deviations are common in young individuals with TCS. However, there are to our knowledge no data on speech in adults or any evaluations of intelligibility in order to measure how well a listener is able to map the acoustic signal onto the intended lexical units [16]. Further there are up to date no reports on speech function associated with the severity of TCS or orofacial functioning.

The objectives of the present study were to explore speech features associated with TCS, and to investigate how these speech characteristics are related to phenotypic severity of the condition.

The specific questions of the present study were:

• How similar are the speech features in TCS in terms of articulation, nasal resonance and velopharyngeal function, voice and intelligibility across ages?

• Are speech characteristics, measured as a speech composite score extracted from findings of articulation errors, nasal resonance and voice aberrations, correlated with phenotypic severity and orofacial function?

• How are structural malformations, like malocclusion, cleft palate and hearing loss, associated with articulation errors, nasal resonance, velopharyngeal dysfunction, altered voice and intelligibility in TCS?

## Methods

### Recruitment

All patients with TCS registered at either the TAKO-centre (National Resource Centre for Oral Health in Rare Medical Conditions), the Department of Medical Genetics and the Centre of Rare Medical Conditions based at Oslo University Hospital, or the Norwegian Craniofacial Association (patient support group), were contacted by mail and offered an extensive health examination focusing on orofacial characteristics and functions including assessment of speech. Twenty-three of the 36 eligible individuals accepted the invitation and all gave informed consent to participate in the present study. Informed consent was obtained from parents of participants below 16 years.

The recruitment procedures and study protocol were approved by the Regional committee for medical research ethics at the University of Oslo (REK Sør-Øst A, approval number S-08553a) as well as the Norwegian Data Inspectorate at Oslo University Hospital (approval reference 05–2008).

### Participants

The study group comprised 19 individuals from 14 families, 13 females and 6 males, geographically spread over ten of the nineteen Norwegian counties. The median age was 34 years (range 5–74). None of the participants fell within the age range 19–29 years. The participants were divided into three subgroups due to potential influence of speech developmental factors: children 5–10 years (n = 4), adolescents 11–18 years (n = 4) and adults from 29 years and older (n = 11). The TCS diagnosis was confirmed by a clinical geneticist [17]. Four of the 23 responding individuals were excluded, two due to unconfirmed TCS diagnosis and two due to an additional congenital neuromuscular condition.

### Data collection

All data were collected within a period of six months. The participants attended clinical examinations during two consecutive days and all assessments were carried out by the same specialists.

### Characteristics of the study group

Data on type of cleft including reconstruction with pharyngoplasty, atresia of the ear canal, hearing, nasal obstruction, narrow hypopharynx including history of tracheostomy, malocclusion, orthodontic treatment, orthognatic surgery, phenotypic expression reflected by TCS severity scores and NOT-S total scores for orofacial dysfunction are presented in Table 1. A full overview of TCS genotype and the individual scorings of phenotypic severity [17] and detailed information on orthodontic treatment need and orofacial dysfunction have been reported earlier [18]. A brief summary is given below to describe the study group.

**Table 1:** Characteristics of the individuals with Treacher Collins syndrome (TCS)

**Partici-pants**	**Type of cleft**^**a**^	**Atresia of the ear canal**^**b**^	**Hearing PTA(dB)**^**c**^	**Nasal obstruction**^**d**^	**Narrow hypopharynx**^**e**^	**Malocclusion**^**f**^	**Orthodontic treatment**	**Orthognatic surgery**^**g**^	**TCS severity scores**^**h**^	**NOT-S total scores**^**i**^
**Adults**										
1	0	0	65.00	0	1	1a	1	0	13	4
2	0	0	13.75	0	0	0	0	0	7	3
3	0	0	68.75	0	1	0	1	0	11	4
4	1	1	67.50	1	1	0	1	0	16	6
5	0	1^^^	56.25	1	1	0	1	1	12	4
6	0	1^^^	95.00	2	1	3a	0	2	15	7
7	0	0	40.00	0	∆	0	0	0	8	4
8	0	2	47.50	0	1	1a	1	0	12	3
9	0	0	26.25	1	0	0	0	0	9	2
10	1*	0	32.50	0	1	1a	0	0	10	7
11	0	0	61.25	1	1	3b	1	0	11	4
**11–18 years**										
12	0	0	53.75	1	1	0	0	0	7	5
13	0	0	32.50	0	1	1b	0	0	7	4
14	2	1	60.00	2	1	0	0	0	17	5
15	0	0	53.75	0	1	0	0	0	12	4
**5–10 years**										
16	0	1	70.00	2	2	2	0	0	15	7
17	1	1	75.00	0	2	2	0	2	16	7
18	2	1	58.75	1	1	0	0	0	14	4
19	0	0	52.50	0	1	1b	0	0	12	4

Present (1), absent (0), missing data (∆).

^a^Isolated cleft palate (1), submucous cleft palate (2), pharyngeal flap (*).

^b^No atresia of the ear canal (0), bilateral atresia (1) and unilateral atresia (2), acquired unilateral facial palsy (^).

^c^Pure-tone average.

^d^No nasal obstruction (0), nasal septum deviation, (1), choanal atresia or stenosis (2).

^e^Narrow hypopharynx (1) including history with tracheostomy (2).

^f^No increased or reverse overjet or open bite (0). Increased overjet > 3.5 to ≤ 6 mm (1a) and > 6 to ≤ 9 mm (1b). Lateral and anterior open bite < 4 mm (2). Reverse overjet > 1 mm to > 3.5 mm (3a) and ≥ 3.5 mm (3b).

^g^No orthognatic surgery (0), LeFort III osteotomy (1), mandibular advancement (2).

^h^Mild TCS phenotype 0–10, severe TCS phenotype 11–20.

^i^ Nordic Orofacial Test-Screening (NOT-S) total score (0–12) reflecting number of affected domains.

An experienced otolaryngologist carried out physical examinations of the head and neck, including flexible nasendoscopy. Five participants had been born with cleft palate and all but one child with a submucous cleft had undergone palatal repair. Pharyngeal flap surgery had been performed in one individual early in adulthood. Both children who had been decannulated after long-term tracheostomy were still using manual signing to augment expressive communication.

Sixteen of 19 participants were using hearing devices. Six had bilateral bone-anchored hearing aids (BAHA) and two had unilateral. Seven had behind-the-ear aids (BTE), five of which were unilateral and two bilateral. One participant was using bilateral in-the-ear hearing aids (ITE). Pure-tone audiometry (ISO 8253–1 1989) was used to update hearing measures. Pure-tone average (PTA) was calculated for the better ear at frequencies of 500, 1000, 2000 and 4000 Hz (M4, World Health Organization). One participant had normal hearing, while four had slight hearing impairment (26–40 dB), seven had moderate (41–60 dB), six had severe (61–80 dB) and one had profound (≥81 dB). The mean PTA dB hearing level was 52.10 (SD 20.04). No differences between age groups or gender were found. Conductive hearing loss was identified in 11 individuals, mixed hearing loss in five and sensorineural loss in two. All except two individuals had appropriately fitted hearing devices. Both were using unilateral hearing aids, but were assessed as requiring bilateral devices.

Data on dental occlusion in terms of increased overjet, lateral and anterior open bite and reverse overjet is given in Table 1. The assessments were made by a specialist in orthodontics according to the Dental Health Component (DHC) grading system [19]. Dental casts supplemented with photographs were used. In two of the cases extremely narrow conditions were hindering taking dental impressions and occlusion was evaluated from photographs only. History of orthodontic treatment and orthognatic surgery were taken from the MHC questionnaire on oral health developed by Mun-H-Center [20] and verified by clinical dental examinations and cephalograms.

TCS severity scores (mild TCS phenotype 0–10, severe TCS phenotype 11–20) were calculated by the clinical geneticist according to a system of quantification of the phenotypic expression in TCS developed by Teber et al. [12]. The median TCS severity score for the present study group was 12 (range 7–17). Hearing loss in terms of mean PTA and TCS severity scores were significantly correlated (rho = 0.75, p < 0.001).

The Nordic Orofacial Test- Screening (NOT-S) was used in assessment of orofacial functions. This protocol contains a structured interview and a clinical examination incorporating six domains with one to five questions or tasks each [21]. The interview domains are sensory function, breathing, habits, chewing and swallowing, drooling, dryness of the mouth, and the examination domains comprises the face at rest, nose breathing, facial expression, masticatory muscle and jaw function, oral motor function and speech. One positive point is given if the level of function is not adequate based on specified criteria generating a total score of 0–12. Facial asymmetry was the most common observation (17/19) present in all but one adult and one adolescent. Breathing problems, mainly snoring, were reported by thirteen of 19 participants (9/11 adults, 3/4 adolescents and 1/4 children). Chewing and swallowing difficulties were equally as frequent (present in 6/11 adults, 3/4 adolescents and all four children). The speech task of counting aloud to ten and fluently repeating ‘pataka’ revealed deviating articulation and/or nasal resonance in six of the eight youngest participants and three of 11 adults.

### Speech samples

Thirteen sentences from a Norwegian translation of Swedish Articulation and Nasality Test (SVANTE) were used to evaluate articulation, nasality and voice [22], see Additional file 1: (a). The sentences were either read aloud following visual presentation on a computer screen, or imitated following verbal presentation by the examiner in cases where the participant was unable to read. Evaluation of intelligibility was based on the speech sample gained from SVANTE’s picture description task (beach scene). In cases where a participant generated less than the required number of 50 words in response to the task, connective utterances from SVANTE’s naming task were added to the sample. For the three adults who generated less than the required 50 words in the picture description task and produced no connected speech during the naming task, conversation from the interview section of the NOT-S assessment of orofacial function was added.

### Speech recordings

Simultaneous digital audio recordings (TASCAM, DR-1, TEAC Corporation, Montebello, CA) and video recordings (Canon Digital Camcorder FS11, Canon U.S.A Inc., Lake Success, NY) were made using an external condenser microphone (Beyerdynamic MCE721, Beyerdynamic GmbH & Co. KG, Heilbronn, Germany). Randomized .wav and .mpeg files were edited in Adobe Premiere 4.0. The sound files were played back using Windows Media Player and the video samples using VLC media player for Windows. Beyerdynamic DT250 headphones were used by the listeners.

### Articulation

Production of consonants was first assessed independently by two speech language pathologists in relation to the target articulations [see Additional file 1: (a)] defined in the SVANTE manual [22]. The perceptual analyses were based on audio recordings of the SVANTE sentences. In consistency with the SVANTE manual, all target consonants were semi-narrowly transcribed, defined as exclusion of aspiration features in this study. Rules for transcription were based on International Phonetic Association conventions (IPA 2005, ExtIPA 1997). Two weeks later, the same observers performed a final consensus assessment transcribing the same target consonants based on video recordings of the speech samples used in the individual assessments.

The consensus assessment formed the basis for a further two-stage categorization of consonant production carried out by the first author. Firstly, the transcriptions of the target consonants were assigned to two binary categories: articulation correct or within normal limits for the region (0), and incorrect articulation (1) when occurring twice or more for each target sound. The results were used for descriptive data on articulatory substitutions and calculation of percentage correct consonants (PCC) [23]. In the second stage of the analysis, the incorrect consonants were classified into six categories of consonant placement errors and cleft palate speech characteristics (CSC) [22,24]. Presence of nasal air leakage (NAL) and weakness were assessed when identified on at least three different sounds [24,25]. The categories and corresponding articulation substitutions are presented in the Additional file 1: (b) [see Additional file 1]. The placement errors are categorized in line with SVANTE’s registration chart [22], and the cleft palate speech characteristics used were those defined by the Scandcleft project [24].

### Nasal resonance and velopharyngeal function

Assessment of nasal resonance was made by three independent speech and language pathologists via perceptual analysis of randomized audio samples of the SVANTE sentences. For both hyper- and hyponasality the median value of the three ratings was reported as the final result for each participant.

Hypernasality was defined as excessive nasal resonance during speech production, resulting from an abnormal coupling of oral and nasal cavities [26]. The rating of hypernasality was based on vowel production in ten of the SVANTE sentences utilizing the modified ordinal 4-point scale [25], see Additional file 1: (c).

Hyponasality was defined as the abnormal reduction or absence of expected nasal resonance associated with nasal consonants [26]. The degree of hyponasality was based on the three SVANTE sentences comprising nasal consonants. Again, an ordinal 4-point scale was used within the rating [see Additional file 1: (c)].

In addition an evaluation of presence or absence of cul de sac resonance was performed by two independent raters. The resonance feature is a variation of hyponasality associated with a muffled tone and considered to be related to blockage of the anterior section of the nasal cavity [26].

Evaluation of velopharyngeal function was based on calculation of the Velopharyngeal Composite Score- Summary (VPC-sum) [24]. This summary outcome measure was calculated by the first author based on the presence of hypernasality, posterior nonoral consonant production, and passive cleft speech characteristics as NAL, and weakness on pressure consonants on three or more target sounds. A score of one was allocated for the presence of deviation on each of the variables. A VPC-sum of 0 or 1 indicates no or only minor VPI, while a VPC-sum of 2 indicates borderline deficiency, and a sum of 3 or 4 indicates insufficient velopharyngeal function.

### Voice

The video recordings of the SVANTE sentences provided the speech samples for the perceptual evaluation of voice characteristics, including deviations of pitch, loudness, flexibility and quality [27]. The evaluation was carried out as a consensus assessment by the same two speech and language pathologists who assessed articulation. Binary ratings of the presence (1) or absence (0) of deviating voice characteristics on the following variables were carried out: altered pitch (low-pitched or high-pitched), altered loudness (decreased or increased intensity), and altered flexibility (monotonous and lacking in energy or increased energy with exaggerated pitch variations when volume and rate increased). Deviating voice quality was described using the variables breathiness, hoarseness, creaky, rough, throaty, restrained, strained and grating [28].

### Intelligibility

Audio recordings of connected speech were used to evaluate the intelligibility of speech in TCS. The recordings were first orthographically transcribed by the first author and subsequently edited to capture the required number of approximately 50 words produced by each of the nineteen participants.

Orthographic transcriptions were used in line with the recommendations by Whitehill [29]. The speech samples were presented in Windows Media Player on a single personal computer (headphones Beyerdynamic DT250) to three naïve listeners for independent transcriptions. All the listeners were healthy native Norwegian adults experienced in working with people with disability, but none of them were acquainted with the participants. Instructions were given to transcribe whole words and mark every unintelligible or incomplete word with an X. It was allowed to pause and rewind the recordings to accommodate the time required for handwriting. The listeners first transcribed the speech samples of the children and adolescents, presented in randomized order, followed by the randomized samples of the adults.

The rating of the orthographic transcriptions was performed by the first author according to the methodology outlined by Hustad et al. [16]. Orthographically transcribed whole words, including misspellings and homophones were evaluated as completely perceived and pooled into the intelligible words category. Words marked with X and incomplete transcriptions were evaluated as not perceived and pooled into unintelligible words. These were supplemented with a third category comprising omitted words when a word was missing compared with the transcriptions of fellow transcribers. The percent of intelligible, unintelligible and omitted words transcribed by the listeners for each speech sample was calculated. The median value of the three ratings was reported as the final result for each participant.

### Speech composite score

Inspired by the composition of a summary outcome measure VPC-sum for velopharyngeal function [24], a speech composite score was constructed by the first author in order to display the variability of deviating speech function concerning articulation, nasal resonance and voice associated with TCS.

The results were extracted from ratings for six speech features investigated. One (1) point indicating speech deviation related to the particular speech feature was given according to the following criteria: 1) Articulation errors, when misarticulation on target consonants occurred at least twice, 2) Nasal air leakage (NAL) and/or Weakness, when observed on three or more consonants, 3) Hypernasality and 4) Hyponasality in case of ordinal rating of 1–3 respectively, and for positive binary ratings of 5) Altered voice quality and 6) Reduced voice intensity and/or flexibility. A point of 0 (zero) indicating no speech deviation was given for negative binary rating concerning articulation errors and the two voice features, and for ordinal rating 0 for hypernasality and hyponasality respectively. The points were added up to provide a speech composite score, ranging from 0–6, indicating an increasing variability of speech impairments.

### Statistical analyses

Statistical analyses of the data were carried out using MedCalc for Windows, version 12.2.1.0 (MedCalc Software, Mariakerke, Belgium). Descriptive statistics were analyzed and given on three age groups (children 5–10 years, adolescents 11–18 years and adults ≥29 years). The Fisher’s exact test was used to test associations between nominal variables. The two younger age groups (n = 4 respectively) were pooled into one group due to small sample sizes. The Mann–Whitney U test was applied to test independence between age groups. Correlation was tested using Spearman’s rank correlation. To reduce the risk of type 1 errors, *p* ≤ 0.01 was interpreted as statistically significant.

### Reliability

Inter- and intrarater agreement of consonant transcription regarding place and manner of articulation, and agreement and transcription of diacritics concerning NAL, were tested by means of percentage agreement, point by point. Interrater reliability between the independent transcribers (audio recordings) and consensus assessment (video recordings) was 88% and 89% respectively for transcription of articulation, and 85% and 89% for diacritics for NAL and weak pressure. Nine of nineteen (47%) randomly selected speech samples were transcribed twice to test intrarater reliability of the consensus assessments of transcriptions. Intrarater agreement for transcription of place and manner of articulation was 96%, while agreement for transcription of NAL was 97%.

Interrater reliability of hypernasality ratings was tested using weighted Kappa statistics and interpreted according to Altman [30]. Kappa values for interrater agreement were K = 0.26-0.55 (i.e. fair to moderate) and intrarater agreement was K = 0.71-0.92 (i.e., good to very good). The same procedure was used for testing reliability of hyponasality ratings. Interrater reliability was K = 0.31-0.57 (i.e. fair to moderate) and Kappa values for intrarater agreement were 0.82-1.00 (i.e. very good). Intertranscriber agreement on percentage of words classed as intelligible, unintelligible and omitted was tested using intraclass correlation (ICC), with absolute agreement. Average ICC was 0.84 for percentage of words classed as intelligible, 0.87 for unintelligible words, and 0.65 for omitted words.

## Results

### Articulation

**Figure 1:**
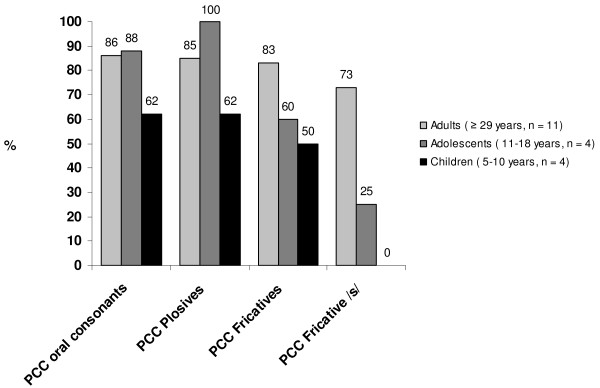
Percentage correct consonants (PCC) in individuals with Treacher Collins syndrome.

Misarticulation of oral consonants was identified in 11/19 participants, all 4 children and 3/4 adolescents and 4/11 adults. The data regarding the percentage of oral consonants articulated correctly is presented in Figure 1 and reveals that plosives were less commonly affected than fricatives in all age groups. The /s/ sound (voiceless fricative dental-alveolar) was clearly the most commonly affected sound (73% correct articulation in adults, 25% in adolescents and 0% in children). No misarticulations of the lateral approximant /l/ were observed.

Consonant placement errors were identified in all age groups and were observed in both the anterior and posterior oral regions (Table 2). The anterior oral placement errors consisted of bilabial production of labiodental fricatives (5), interdental production of bilabial plosives (2), and labiodental production of bilabial plosives (1). Lateral production of /s/ occurred in seven cases, and three children had interdental, palatal or velar production of the /s/ sound. Retraction of plosives to uvular position was the most frequent posterior oral error and was observed in 4 cases. Four participants, two children and two adults, were assessed with both velar/uvular articulation errors and anterior misarticulations.

**Table 2:** Distribution of consonant placement errors and cleft palate characteristics in individuals with Treacher Collins syndrome (n = 19)

	**Anterior oral**	**Posterior oral**	**Posterior nonoral**	**Lateral /s/**	**Nasal Air Leakage**	**Weakness**
Adults (n = 11)	3	3	0	3	3	2
11-18 years (n = 4)	2	1	0	3	2	2
5-10 years (n = 4)	3	2	0	1	3	3
**Total**	**8**	**6**	**0**	**7**	**8**	**7**

Passive cleft speech characteristics of NAL were identified in 8 participants, and weak pressure consonants were exhibited in production of voiced plosives by 7 of them (Table 2).

### Nasal resonance and velopharyngeal function

**Figure 2:**
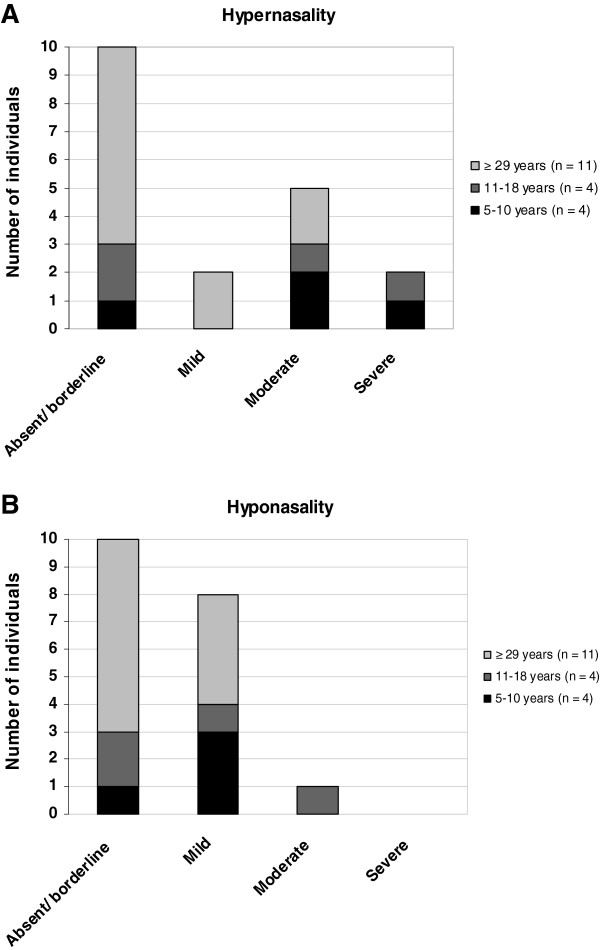
Occurrence of hypernasality (A) and hyponasality (B) in individuals with Treacher Collins syndrome.

Hypernasality was observed in 9/19 participants, and occurred more frequently and more severely in children and adolescents than in adults, but no significant difference was found. (Figure 2A). The median score was 0 (range 0–2) for the adults, median 1 (range 0–3) for the adolescents and median 2 (range 0–3) among the children.

Mild hyponasality was identified in 8/19 participants and moderate hyponasality in one of the adolescents (median 0, range 0–2) (Figure 2B). The possible relationship between hyponasality and nasal malformations was tested, but no significant association was found. Both hyper- and hyponasality (mixed nasality) was observed in six individuals, three of whom had a history of isolated cleft palate.

Cul de sac resonance was assessed in two of the adolescent, both identified with need for correction of nasal obstruction at the examination performed during the present study.

Five participants, three children, one adolescent and one adult, obtained a VPC-sum of 3 indicating VPI. Borderline VPI (VPC-sum 2) was identified in one individual.

### Voice

Deviating voice characteristics were observed in 12/19 cases and were present in all children and adolescents. None of the male adults presented with altered voice, while 4 adult females showed voice aberrations. Hoarseness in combination with a creaky or grating voice was perceived in all of them, and these deviations were also identified in 5 of the children and adolescents. Breathy, hyperfunctional and restrained voice quality was observed in one adolescent. Decreased intensity was identified in all four adolescents, three of whom also presented with reduced vocal flexibility. Two of the adults had altered pitch; one low-pitched and one high-pitched.

### Intelligibility

The median percentages of intelligible and unintelligible words were significantly higher in the adult group than among children and adolescents (Table 3). Two of the adolescents had results comparable with those of the adults, with a minimum of 96% and a maximum of 100% of words transcribed as intelligible by all the transcribers. Three of the four youngest participants presented with the lowest median percentages of intelligible words with 31%, 59% and 67% respectively. These three speech samples were clearly the most inconsistently transcribed. Among the participants with the highest percentage of intelligible words only sporadic words were missing or marked as unintelligible, while longer sequences of words surrounding the unintelligible words typically were missing in the transcriptions of the children with the lowest percentage of intelligible words.

**Table 3:** Percent of intelligible, unintelligible and omitted words in connected speech in individuals with Treacher Collins syndrome

**Word category**	**Adults**	**5-18 years**	**Mann–Whitney U test**	
	**(n = 11)**	**(n = 8)**		
	Median % (range)	Median % (range)	**U**	**p**
Percent of intelligible words	98 (93–100)	77 (31–99)	14.5	0.01
Percent of unintelligible words	0 (0–3)	6 (0–26)	13.0	0.006
Percent of omitted words	2 (0–7)	14 (0–27)	16.0	0.02

### Speech composite score

The speech composite scores (0–6) are presented in Table 4. Three of 11 adults and 6/8 children and adolescents were identified with four or more speech deviations. Seven of the adults presented with no or one single deviating speech characteristic. No significant difference between age groups was established (U = 16.00, p = 0.02). The variability of speech dysfunction was exhibited by individual combinations of speech deviations in 13/19 participants. The speech composite scores were significantly correlated with the TCS severity scores (rho = 0.56, p = 0.01) and the NOT-S total scores (rho = 0.74, p < 0.001).

**Table 4:** Speech composite scores of individuals with Treacher Collins syndrome (n = 19)

**Partici-pants**	**Articulation errors**^**a**^	NAL^*^and/or Weakness^**a**^	**Hypernasality**^**b**^	**Hypernasality**^**b**^	**Altered voice quality**^**a**^	**Reduced voice intensity and/or flexibility**^**a**^	**Speech composite score**^**c**^
**Adults**							
1	1	0	0	0	0	0	**1**
2	0	0	0	1	0	0	**1**
3	0	0	0	0	1	0	**1**
4	1	1	1	1	1	0	**5**
5	0	0	1	0	0	0	**1**
6	1	1	1	1	0	0	**4**
7	0	0	0	0	0	0	**0**
8	0	0	0	0	0	0	**0**
9	0	0	0	0	0	0	**0**
10	1	1	0	1	1	0	**4**
11	0	0	1	0	1	0	**2**
**11-18 years**							
12	1	0	1	1	0	1	**4**
13	1	0	0	0	1	1	**3**
14	1	1	1	1	1	1	**6**
15	0	0	0	0	0	1	**1**
**5-10 years**							
16	1	1	0	1	0	1	**4**
17	1	1	1	1	1	0	**5**
18	1	1	1	0	1	0	**4**
19	1	1	1	1	1	0	**5**
	**11**	**8**	**9**	**9**	**9**	**5**	

^a^Presence (1) or absence (0) of deviating speech feature.

^b^Zero (0) points for ordinal rating of 0 and one (1) point for ordinal rating of 1–3.

^c^Summary score of 0–6 reflecting number of affected speech features.

^*^Nasal Air Leakage.

### Associations between structural malformations, hearing loss and speech

The different types of substitutions identified in individuals with malocclusion in terms of lateral and anterior open bite, increased overjet and reverse overjet are presented in Table 5. A significant association was found between speech composite scores higher than 4 and cleft palate (p = 0.01). Significant correlation was found between hearing loss (PTA) and VPC-sum (rho = 0.57, p = 0.01). This was the only association between any speech feature and hearing impairment.

**Table 5:** Type of substitutions identified in individuals with different types of occlusion in Treacher Collins syndrome (n = 19)

	**Increased overjet**	**Reverse overjet**	**Anterior and lateral open bite**	**No malocclusion**	**Number of individuals**
	**(n = 5)**	**(n = 2)**	**(n = 2)**	**(n = 10)**	**(n = 19)**
**Anterior oral substitutions**	**4**	**1**	**2**	**2**	**8**
Bilabial articulation	2	0	1	1	4
Labiodental articulation	1	0	0	0	1
Interdental articulation	1	0	1	0	2
Lateral /s/	4	1	0	2	7
**Posterior oral substitutions**	**2**	**1**	**1**	**2**	**6**
Palatal articulation	0	0	0	1	1
Retracted to velar	0	0	1	0	2
Retracted to uvular	2	1	0	1	4
Double articulation	1	0	0	0	1
**No articulation errors**	**1**	**1**	**0**	**6**	**8**

## Discussion

The primary objectives of the study were to explore the speech characteristics associated with TCS in different age groups, and to investigate if speech function was correlated with phenotypic severity of the condition. Speech composite scores, including six speech features were extrapolated from the results of the articulation, nasal resonance and voice assessments. Most of the adults presented with maximum one affected speech feature, while all but one of the children and adolescents displayed multiple deviations. Intelligibility was clearly reduced among the youngest participants. Speech composite scores were significantly correlated with TCS severity scores and NOT-S scores measuring orofacial dysfunction.

Articulation errors were observed in almost all of the children and adolescents, and seem to persist in some adults with TCS. Size and shape of the oral, nasal and pharyngeal cavities improved by orthodontic treatment and extensive surgery performed after ended skeletal growth in late adolescence, may have contributed to improved speech in the older participants. The percent correct consonants (PCC) among the children was 62%, which is slightly below the mean of 69% correct consonants reported among primary-school aged children with hearing loss of least 40 dB HL [31]. The corresponding values for 5 and 7 years old children with unilateral cleft lip and palate were 82.8% and 81.0%, while healthy non-cleft comparisons had PCC’s of 100% and 98% respectively [32].

The /s/ sound was the most commonly misarticulated speech sound in all groups, with lateral production more frequent than interdental. These findings contradict those of Vallino-Napoli who observed a greater proportion of interdentalization [11]. In line with earlier findings were bilabial and interdental productions mainly observed visually [15] when analyzing the video recordings, but not detected on the audio samples and therefore unlikely to reduce intelligibility.

Anterior oral and posterior oral placement errors occurring with similar frequency seem almost exclusively to be related to structural malformations since only one of the children was assessed to have a combined phonologic and dyspraxic disorder. In this case backing of dental sound coexisted with final consonant deletion, syllable reduction, difficulties in the perception and imitation of speech and limited tongue mobility.

It has previously been suggested that articulation errors in TCS are associated with anterior open bite malocclusion [10,11] and retroglossia [10]. In the current study malocclusion, mainly increased maxillary overjet, occurred in nine participants. All the three adults identified with articulation errors had malocclusion. Posterior oral articulatory displacements outside the occlusional frame, i.e. uvular articulation, may have been related to a cleft palate or a disproportion between narrowed oral cavity caused by hypoplasia of the mandible and normal sized tongue [17,18]. Further research using electropalatography may contribute to better understanding of articulatory movement patterns in TCS.

No posterior nonoral placement errors were detected, but one of the children with cleft palate had a history of pharyngeal and glottal replacements which had recently been addressed in speech therapy. In clinical experience this type of compensatory articulation sometimes becomes evident early in speech development in children with TCS without clefts. Referral for further investigation of the soft palate and velopharyngeal function is indicatory for appropriate speech therapy planning.

Hyponasality identified in nearly half of the cases, was as prevalent as hypernasality, but occurred in a milder degree. Two of the participants with hyponasality were assessed with cul de sac resonance as well as a muffled resonance. Both of them were in need of nasal reconstruction due to choanal atresia or severe nasal septum deviation. However, the suggested association between reduced nasal resonance and nasal malformations was not confirmed in the current study [10,11].

Hearing loss in TCS is mainly conductive, originating from malformations of the outer and middle ear. The correlation between the level of hearing loss and TCS severity scores in the study group was strong. Considering that the majority of participants had moderate to severe hearing impairment one would expect an influence on speech. The only significant association, however, was between mean PTA and VPC-sum indicating VPI. This association implies that both hearing loss and velopharyngeal dysfunction most likely will be found in severely affected individuals.

In clinical experience, toddlers with TCS are dependent on continuous use of hearing aids to elicit language development and communication skills. The current study group mainly comprised teenagers and adults, who to our understanding, had been using hearing aids since childhood. Hearing loss may be of less importance for speech production after the articulation skills have been established. Longitudinal follow-up of developing children with TCS could reveal the effects of hearing loss on speech acquisition.

One of the adults had previously undergone pharyngoplasty to resolve VPI and was now assessed with mixed nasality. Four children and adolescents with VPI had obstructive sleep apnea (OSAS) caused by airway restrictions [17]. Pharyngeal flap surgery constricts the upper airway and may contribute to upper air way resistance and aggravate sleep apnea. Pharyngeal flap surgery was therefore considered contraindicated.

The resonance in TCS has been characterized as unusual and muffled [16]. This type of resonance relates to elevation and retraction of the tongue assuming to lead to further constriction of the oropharynx and subsequent damping and vowel distortion [33]. Without having explicitly defined criteria and assessed muffled resonance, Vallino-Napoli estimated that forty percent of the resonance aberrations in a sample of children and adolescents were muffled [11]. In the present study two experienced listeners were independently analyzing the speech samples perceptually twice in order to capture this unusual resonance. Eventually aberrant resonance features affecting the vowels were identified in two adults and one child. All of them also had deviant nasal resonance and pronounced retraction of the articulation. We concluded that it was impossible to determine whether this was an articulatory or resonance feature, and therefore not reported as results. Due to lack of a distinct definition of muffled resonance, future studies using instrumental analyses are essential to determine the acoustic correlates and clearly define muffled resonance. Assessment of patients with various congenital conditions with narrow hypopharynx and retroglossia, like Pierre Robin sequence and Weaver syndrome, would contribute to information of prevalence and clinical significance of this feature.

It has been suggested that clinicians often experience difficulty to assess nasal resonance in TCS due to obstruction of the nasopharyngeal and oropharyngeal airway [15]. This may also have contributed to the variation in interrater judgments of nasal resonance in the present study. However, analyses of the results show that there was perfect agreement on absence of hypernasality in the children. The decision to take the median grading of the three raters as the result effectively eliminated outliers and the results for the children thereby appear fairly reliable. Regarding hyponasality there was perfect intrarater agreement on the rating of nine of the eleven adults, but greater disagreement in ratings of the younger participants. In future studies, supplementation of perceptual analyses with nasometry is recommended to improve the accuracy of the assessments of nasal resonance [34].

Hoarse, grating and creaky voice quality was identified using perceptual analysis in nearly half of the participants and in all five patients with clefts. This may be related to compensatory muscular strain used for voice production due to altered resonance cavities [11]. In addition to altered voice were frequent attempts to moisture the lips and palate by licking immediately before speech onset and between longer utterances observed. Subtle audible sounds of friction or clicking were noticed at tongue release from the palate during articulatory movements. It was hypothesized that some of the voice characteristics of TCS may be related to reduced mucosal lubrication associated with low unstimulated salivary secretion rate identified in nearly half of the study group [35]. Yet no significant association between these factors was found. It would be interesting to explore this hypothesis further by using instrumental analysis methods on a larger TCS sample with more detailed examination of the impact of salivary secretion on voice and articulatory function.

Decreased voice intensity and flexibility with reduced vocal energy was observed in three of the adolescents. A few of the adults presented with elements of these voice characteristics, but were not judged to be dysfunctional. The voice characteristics seemed to create a suprasegmental layer over other speech characteristics and may be further enhanced by sound distortions produced by constrictions at all levels of the pharynx [8]. Two of these three adolescents presented with sensorineural hearing loss and this may have influenced on voice and speech outcome since aberrant prosodic modulation has been reported in association with this type of loss [36].

The speech of the adults was almost completely intelligible to naïve listeners suggesting that their speech impairments had a minor impact on participation in social interaction. The variability of speech intelligibility among younger participants, however, was considerably larger with variation from performance on the level of the adults to a percentage of intelligible words below 50%. The inconsistency and the median of intelligible words among the children with TCS was clearly divergent from the approximately 80-90% depending of utterance length found in typically developing four year old children [16].

The fact that the proportion of omitted words was larger than unintelligible words in both age groups was puzzling. A discussion with the three transcribers of the intelligibility evaluation revealed a few interesting aspects. All agreed that the transcription task was easier than expected and most of the individuals were easy to understand. However, all transcribers had noticed that when the speech rate accelerated the words became difficult to detect; and they became uncertain if they had marked the correct number of words. All listeners had problems comprehending some of the children, but not necessarily the same ones. They reported individual weaknesses in perceiving certain types of speech features like hypernasality, dialect traits and articulation errors. This underlines that successful communication is not only dependent on the speech functions of the speaker, but also on the communication skills of the listener. There is some knowledge of how adolescents with TCS learn to cope with the reactions of others [37]. However, the potential challenges in communicative participation in variable social and environmental contexts faced by a broader population have not yet been explored.

The low percentage of intelligible words among the youngest participants underlines the need to facilitate speech and communication skills. Considering the high prevalence of deviating speech features and hearing loss, early referral for assessment of speech function and communication is recommended. Early introduction of sign language is often useful to augment communication and language development. One should recognize that the speech composite scores were correlated with the TCS severity scores and the NOT-S scores, which express the close inter-relationship between structural malformations and different orofacial functions [18]. It is important that clinicians realize that identification of speech aberrations in individuals of all ages with TCS is a clear indication to perform assessments of respiration and food intake and vice versa.

Delayed speech development is one of the items in the severity rating system created by Teber et al. and was reported in a majority of the individuals [12]. Unfortunately there was no description of the criteria or data collection procedures used. The reliability of using speech delay as an item is questionable when one considers the difficulties in defining valid criteria and collecting data. The speech aspect in evaluation of TCS phenotype may change over time and should therefore be based on a current status. The set of speech features captured by the speech composite score appears to reflect the phenotypic variability in one functional aspect of TCS.

A comprehensive multidisciplinary protocol outlined by Thompson et al. involving systematic follow-up and treatment planning during maturation has the potential to contribute to improved outcomes in terms of speech and communication, respiration, hearing, and orthodontic status [10]. The present study showed that a subgroup of adults with TCS may require multidisciplinary health services due to persisting speech deficits. Sleep apnea was frequent among the adults and due to expected changes over time regular follow-up was suggested [17]. Reduced salivary secretion was also common and is a risk factor for oral disease [35]. Based on these findings one can assume that ageing individuals with TCS may be at higher risk for developing health problems. In order to acquire a genuine understanding of the associations between the overall severity of the syndrome, orofacial dysfunction, hearing impairment and speech development, a prospective longitudinal study is needed.

### Advantages and limitations of the study

Established speech assessment methods have been used to describe speech function. The speech assessment methods have been accurately described in order to ensure reproducibility and make comparisons with larger cohorts possible. Further the speech findings have been related to other features of the same study group to provide better understanding of phenotypic expression of TCS. Interpretation of the findings must be careful due to limited sample size and number of test performed.

Unfortunately this study has failed to investigate what factors the children and their parents considered to be limiting in communication. There is clearly a need of a thorough study using an extensive communication questionnaire and in depth interviews to identify the factors that support or undermine communication skills in children and adolescents with TCS. Furthermore, this study only gives an impression of the speech problems at different ages.

## Conclusions

In conclusion this study showed that children, adolescents and a subgroup of adults had multiple aberrations of articulation, resonance and voice features. Only children presented with markedly reduced intelligibility. Speech dysfunction was significantly correlated with phenotypic severity of TCS. The complexity of speech problems identified in young individuals with TCS indicate that speech monitoring and intervention are required from early in life preferably in multidisciplinary craniofacial team settings. A subgroup of adults with persisting speech deviations may require prolonged attention of speech language pathologists.

## Abbreviations

BAHAm: Bone-anchored hearing aids; BTE: Behind-the-ear hearing aids; CSC: Cleft palate speech characteristics; DHC: Dental Health Component; IPA: International Phonetic Association; ITE: In-the-ear hearing aids; PCC: Percentage correct consonants; NAL: Nasal air leakage; NOT-S: Nordic Orofacial Test- Screening; PTA: Pure-tone average; SVANTE: Swedish Articulation and Nasality Test; TCS: Treacher Collins syndrome; VPC-sum: Velopharyngeal Composite Score- Summary; VPI: Velopharyngeal insufficiency.

## Competing interests

The authors declare that they have no competing interests.

## Authors’ contributions

PÅ and CP created the project outline regarding this part of the collaborative project. PÅ acquired the necessary approvals, coordinated the examinations, performed data collection, and wrote the main body of the manuscript draft. CP has taken part in some of the speech analyses and made valuable contributions to the manuscript in her role as supervisor of the master thesis of PÅ. HA contributed with the assessments of hearing and critically revised the manuscript drafts. All collaborators have approved the final manuscript.

## Pre-publication history

The pre-publication history for this paper can be accessed here:

http://www.biomedcentral.com/1471-2350/15/47/prepub

## Supplementary Material

Additional file 1 Materials for speech assessments. Click here for file
