# Supplementary material for: Associations between speech features and phenotypic severity in Treacher Collins syndrome
Source: BMC Med Genet. 2014 Apr 28;15:47. doi: 10.1186/1471-2350-15-47 (PMC4101868; doi:10.1186/1471-2350-15-47)
Supplement: Additional file 1 — Materials for speech assessments. [file 1471-2350-15-47-S1.pdf]

Additional file 1
Materials for speech assessments

a. Sentences in Swedish Articulation and Nasality Test (SVANTE), 2005

Swedish	Norwegian translation	Transcription of target consonants	English translation of Norwegian version
Pippis apa piper	Pippis ape piper	p	Pippi's monkey is whining
Bibbi bara jobbar	Bibbi bare jobber	b (○)	Bibbi is just working
Titti tittar på TV	Titti titter på TV	t	Titti is watching TV
David och du leder	David og du leder	d (○)	David and you are leading
Kicki kokar korv	Kikki baker kaker	k	Kicki is boiling sausage
Giggi vill väga guld	Giggi vil lage gull	g (○)	Giggi wants to make gold
Sissi å Lasse sover	Sissi og Lasse sover	s	Sissi and Lasse are asleep
Fiffi får kaffe	Fiffi får kaffe	f	Fiffi gets coffee
Vivvi vevar	Vivi vever	v	Vivvi is weaving
Lollo lurar Ella	Lollo lurer Ella	l	Lollo is deluding Ella
Svante vill inte ha vantar	Svante henter ikke votter	n	Svante doesn't get gloves
Anki hämtar hinken	Anki hopper og hinker	ŋ	Anki jumps and hops
Mimmi å mamma e hemma	Mimmi og mamma er hjemme	m	Mimmi and mum are at home

b. Categories of consonant placement errors and cleft palate speech characteristics

Anterior oral	Posterior oral	Posterior nonoral	Lateral production	Nasal Air Leakage	Weakness
Bilabial	Palatal Velar	Pharyngeal plosive	Lateral /s/	Nasal friction	Weak pressure
Linguolabial	Uvular	Pharyngeal fricative		Velopharyngeal	consonants
Interdental	Velar/uvular	Glottal plosive		friction	Nasalized voiced
Labiodental	double arti-	Glottal double-		Fistula friction	pressure
Dentolabial	culation	articulation			consonants
Dental alveolar		Nasal fricative			

c. Rating scales for hypernasality and hyponasality

**Hypernasality**<br>**Score**	**Rating**	**Criteria**
0	Absent or borderline	Nasal resonance is within normal limits for the region or with some perceptible increase in nasal resonance
1	Mild	Hypernasality is evident on close vowels
2	Moderate	Hypernasality is evident on both close and open vowels
3	Severe	Hypernasality is evident on vowel productions and voiced consonants
**Hyponasality**<br>**Score**	**Rating**	**Criteria**
0	Absent or borderline	Nasality was within normal limits for the region
1	Mild	Mildly denasalized nasal consonants
2	Moderate	Markedly denasalized nasal consonants
3	Severe	Almost completely denasalized nasal consonants
